# Nationwide Glaucoma incidence in end stage renal disease patients and kidney transplant recipients

**DOI:** 10.1038/s41598-021-86846-3

**Published:** 2021-04-01

**Authors:** Jong Joo Moon, Yong Woo Kim, Baek-Lok Oh, Kyungdo Han, Dong Ki Kim, Kwon Wook Joo, Yon Su Kim, Ki Ho Park, Hajeong Lee, Yong Chul Kim, Jin Wook Jeoung

**Affiliations:** 1 Division of Nephrology, Department of Internal Medicine, Seoul National University Hospital, 101 Daehak-ro, Jongno-gu, Seoul, 03080 Korea; 2 Department of Ophthalmology, Seoul National University Hospital, 101 Daehak-ro, Jongno-gu, Seoul, 03080 Korea; 3 Department of Medical Statistics, College of Medicine, Catholic University of Korea, Seoul, Korea

**Keywords:** Chronic kidney disease, Optic nerve diseases, Epidemiology

## Abstract

Glaucoma shares common risk factors with chronic kidney disease (CKD) but previous cross-sectional studies have demonstrated discrepancies in the risk of glaucoma in CKD patients. This study enrolled kidney transplantation recipients (KTRs) (n = 10,955), end stage renal disease (ESRD) patients (n = 10,955) and healthy controls (n = 10,955) from National Health Insurance Service database of the Republic of Korea. A Cox proportional hazard regression model was used to calculate the hazard ratios (HR) for primary open-angle glaucoma (POAG) and primary angle-closure glaucoma (PACG) incidences. The incidence of POAG was higher in ESRD patients (3.36/1,000 person-years, *P* < 0.0001) and KTRs (3.22 /1,000 person-years, *P* < 0.0001), than in healthy controls (1.20/1,000 person-years). However, POAG risk showed no significant increase in either ESRD patients (*P* = 0.07) or KTRs (*P* = 0.08) when adjusted for the confounding factors. The incidence of PACG was significantly higher in ESRD patients (0.41/1,000 person-years) than in healthy controls (0.14/1,000 person-years, *P* = 0.008). The PACG incidence was significantly lower in KTRs than in ESRD patients (HR = 0.35, *P* = 0.015). In conclusion, this nationwide cohort study demonstrated that kidney transplantation can reduce the risk of PACG but not POAG in ESRD patients.

## Introduction

Glaucoma is one of the leading causes of irreversible blindness, and the number of glaucoma patients is estimated to increase to 111.8 million by 2040^1,2^. The worldwide prevalence of primary open-angle glaucoma (POAG) and primary angle-closure glaucoma (PACG) is estimated to be 3.54% and 1.09%, respectively^2^. Although PACG is estimated to affect approximately 26% of the entire glaucoma population, it is responsible for nearly half the cases of glaucoma-related blindness worldwide^1^. Considering that Asia accounts for approximately 60% of world’s population and has a rapidly aging society, the burden of glaucoma is expected to increase disproportionately in Asia^2,3^.

Chronic kidney disease (CKD) is another growing public health problem that affects between 8 and 16% of the population worldwide^4^. POAG shares common risk factors with CKD, including old age, hypertension (HTN), and diabetes mellitus (DM)^5^. However, previous cross-sectional studies have demonstrated discrepancies in the risk of POAG in CKD patients. Some studies have reported a greater risk of glaucoma or suspected cases of glaucoma in CKD patients than in those without CKD^6,7^, whereas other studies have reported no significant associations between CKD and POAG when adjusted for multiple confounding factors^8,9^. The progression of CKD eventually leads to end stage renal disease (ESRD), which requires renal replacement therapy for survival. The adverse outcome of ESRD compared to those of the early stages of CKD warrants further investigation to ascertain whether POAG risk increases in ESRD patients.

Kidney transplantation (KT) is the most effective and preferred modality among the renal replacement therapies for ESRD patients^10^. Dialysis—another treatment option for ESRD—is known to induce fluctuations of blood pressure (BP) or intraocular pressure (IOP), which may further elevate POAG risk^11^. In addition, dialysis can induce the shallowing of the anterior chamber depth^12–14^ and can cause acute angle-closure attack^15^, which increases PACG risk. However, to the best of our knowledge, there is limited evidence on the incidence of PACG in ESRD patients. KT not only improves the renal function, but also eliminates the risk of dialysis induced IOP/BP fluctuation and anterior chamber shallowing; thus, further studies are needed to investigate whether KT can reduce the risk of POAG or PACG in ESRD patients.

Therefore, the present study was initiated to investigate the following: (1) to identify glaucoma (POAG and/or PACG) incidence in ESRD patients compared to that in subjects with healthy kidney function; and (2) to evaluate nationwide population-based cohorts in Korea to ascertain whether KT can reduce glaucoma (POAG and/or PACG) risk in ESRD patients.

## Results

### Subject demographics

The mean age of the subjects was 45.8 ± 10.5 years and 59.2% of subjects were men. There was a significant difference in income among the study groups (*P* < 0.0001). A larger proportion of KTRs and ESRD patients had a history of underlying chronic disease such as DM, HTN, and dyslipidemia when compared with healthy control (all *P*s < 0.0001). Among the ESRD patients, 74.1% of the patients underwent hemodialysis, 20.5% underwent peritoneal dialysis, and 5.4% underwent mixed dialysis. Among the KTRs, 31.8% had no dialysis history, 44.6% underwent hemodialysis, and 17.1% underwent peritoneal dialysis before KT. For KTRs, 94.8% of the recipients received induction medication (anti-thymocyte globulin, 8.4%; basiliximab, 86.4%). Almost all the KTRs (97.6%) were prescribed calcineurin inhibitor for maintenance therapy (tacrolimus, 81.6%; cyclosporin, 16.0%) and 15.6% of the KTRs experienced desensitization. The KTRs took postoperative immunosuppressant and corticosteroid as a maintenance therapy to prevent graft rejection. The baseline characteristics of each group are provided in Table 1.

**Table 1:** Subject demographics.

	Healthy control (n = 10,955)	ESRD (n = 10,955)	KTR (n = 10,955)	*P*-value
**Age, years (%)**	45.8 ± 10.5	45.8 ± 10.5	45.8 ± 10.5	1^a^
20–29	782 (7.28)	782 (7.28)	782 (7.28)	
30–39	2,294 (20.94)	2,294 (20.94)	2,294 (20.94)	
40–49	3,529 (32.21)	3,529 (32.21)	3,529 (32.21)	
50–59	3,303 (30.15)	3,303 (30.15)	3,303 (30.15)	
60–69	981 (8.95)	981 (8.95)	981 (8.95)	
≥ 70	50 (0.46)	50 (0.46)	50 (0.46)	
Male sex, n (%)	6,484 (59.19)	6,484 (59.19)	6,484 (59.19)	1^b^
Diabetes mellitus, n (%)	692 (6.32)	4,416 (40.31)	4,416 (40.31)	< 0.0001^b^
Hypertension, n (%)	1,796 (16.39)	10,003 (91.31)	10,003 (91.31)	< 0.0001^b^
Dyslipidemia, n (%)	1,286 (11.74)	4,667 (42.6)	6,170 (56.32)	< 0.0001^b^
**Income, quartile, n (%)**				< 0.0001^b^
Aid	271 (2.47)	2,524 (23.04)	1,580 (14.42)	
Q1	2,828 (25.81)	3,021(27.58)	2,203 (20.11)	
Q2	2692 (24.57)	2,300 (20.99)	2,156 (19.68)	
Q3	2,555 (23.32)	1,814 (16.56)	2,296 (20.96)	
Q4	2,609 (23.82)	1,296 (11.83)	2,720 (24.83)	
**Dialysis modality, n (%)**				< 0.001^b^
No Dialysis History	10,955 (100)	0 (0)	3,487 (31.83)	
Hemodialysis	0 (0)	8,119 (74.11)	4,890 (44.64)	
Peritoneal dialysis	0 (0)	2,242 (20.47)	1,878 (17.14)	
Mixed dialysis	0 (0)	594 (5.42)	700 (6.39)	
**Dialysis duration, years**	0 ± 0	2.86 ± 3.03	2.82 ± 3.21	< 0.0001^c^
< 3 months	10,955 (100)	2,255 (20.58)	3,551 (32.41)	< 0.0001^b^
3 months-1 year	0 (0)	1,831 (16.71)	1,384 (12.63)	
1–2 years	0 (0)	1,706 (15.57)	1,014 (9.26)	
2–3 years	0 (0)	1,150 (10.5)	811 (7.4)	
3–4 years	0 (0)	864 (7.89)	690 (6.3)	
4–5 years	0 (0)	690 (6.3)	762 (6.96)	
> 5 years	0 (0)	2,459 (22.45)	2,743 (25.04)	
**Induction medication, n (%)**				< 0.0001^b^
No use	10,955 (100)	10,955 (100)	574 (5.24)	
Antithymocyte glubulin	0 (0)	0 (0)	920 (8.4)	
Baxiliximab	0 (0)	0 (0)	9,461 (86.36)	
**Desensitization, n (%)**				< 0.0001^b^
No	10,955 (100)	10,955 (100)	9,252 (84.45)	
Yes	0 (0)	0 (0)	1,703 (15.55)	
**CNI for maintenance therapy, n (%)**				< 0.0001^b^
None	10,955 (100)	10,955 (100)	263 (2.4)	
Tacrolimus	0 (0)	0 (0)	8,937 (81.58)	
Cyclosporin	0 (0)	0 (0)	1,755 (16.02)	

Mean ± standard deviation.

*ESRD* end stage renal disease,* KTR* kidney transplantation recipients, *CNI* calcineurin inhibitor.

^a^Comparison was performed using ANOVA.

^b^Comparison was performed using chi-square test.

^c^Comparison was performed using the Kruskal–Wallis test due to absence of normality.

### POAG incidence in KTRs and ESRD patients

**Figure 1:**
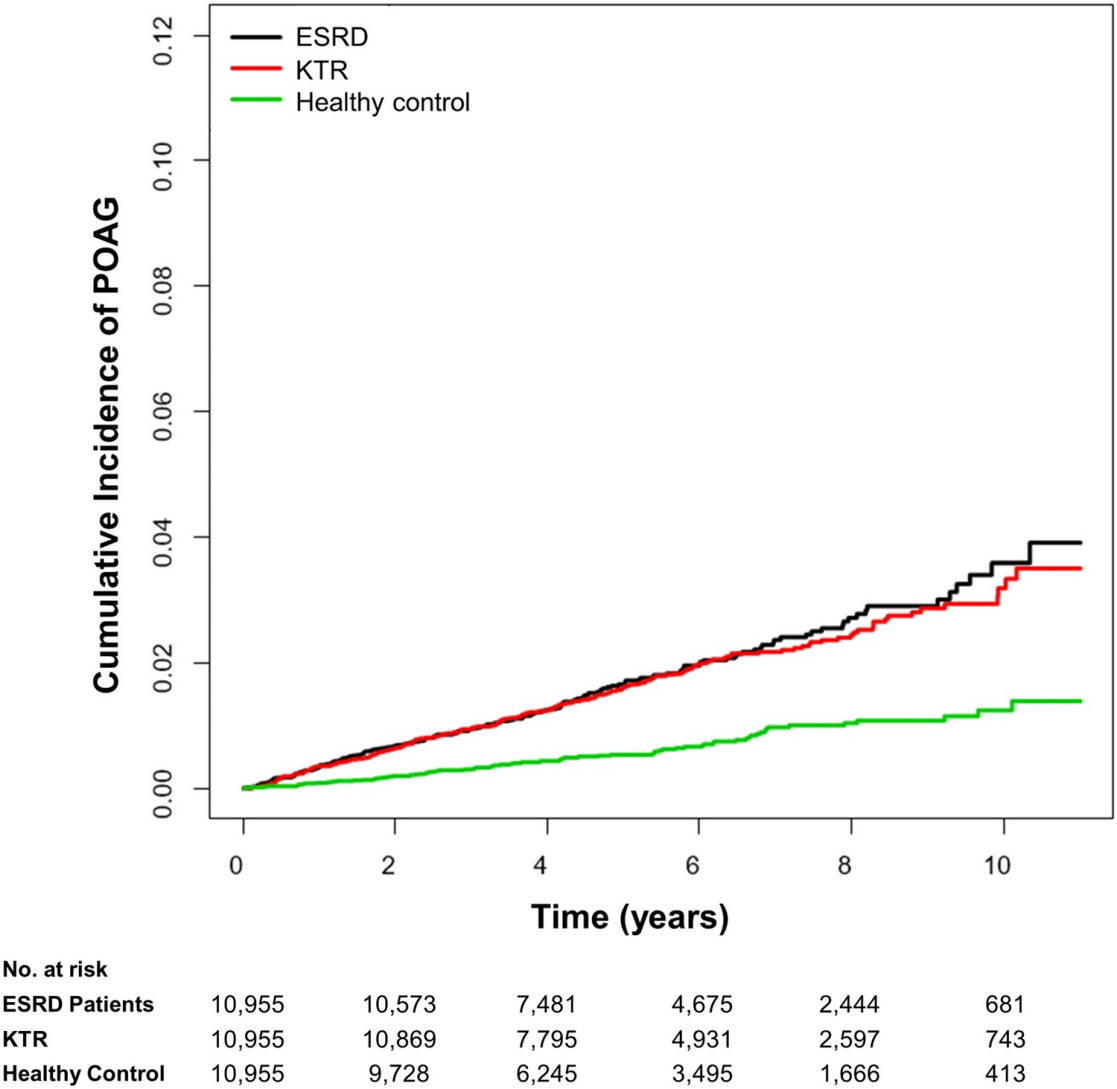
Cumulative Incidence of POAG in ESRD Patients, KTRs, and Healthy Controls. POAG incidence was significantly greater in ESRD patients (3.36/1,000 person-years, *P* < 0.001) and KTRs (3.22 /1,000 person-years, *P* < 0.001) as compared to healthy controls (1.20/1,000 person-years). However, there were no significant increase of risks for POAG incidence in neither ESRD patients (*P* = 0.07) nor KTRs (*P* = 0.08) when adjusted for age, sex, DM, HTN, dyslipidemia, income, and CCI. There was no significant difference in POAG incidence rates between ESRD patients and KTRs. *KTR* kidney transplant recipient, *KT* kidney transplant, *CKD* chronic kidney disease, *ESRD* end stage renal disease, *POAG* primary open-angle glaucoma, *DM* diabetes mellitus, *HTN* hypertension, *CCI* Charlson comorbidity index.

During the study period, a total of 457 patients (1.4%) were diagnosed with POAG. The incidence rate of POAG was significantly greater among ESRD patients (3.36/1,000 person-years, *P* < 0.0001) and KTRs (3.22 /1,000 person-years, *P* < 0.0001), compared with healthy controls (1.20/1,000 person-years) (Fig. 1). When adjusted for age and sex (Model 1), ESRD patients (HR = 2.95, *P* < 0.0001) and KTRs (HR = 2.72, *P* < 0.0001) had increased risk of POAG than healthy controls (Table 2). However, there were no significant increase in the risks of POAG neither in ESRD patients (*P* = 0.07) nor KTRs (*P* = 0.08) when adjusted for age, sex, DM, HTN, dyslipidemia, income, and CCI (Model 2). There was no significant difference in POAG incidence rates between ESRD patients and KTRs (Table 2). The numbers of subjects at risk of POAG among the study groups, stratified by time, are provided (Supplementary Table 1). The subgroup analysis according to the confounding variables including age, sex, DM, HTN, dyslipidemia, and durations of dialysis are provided in Supplementary Table 2.

**Table 2:** POAG incidence in ESRD patients, KTRs, and healthy controls.

Group	N	POAG Incidence	Duration (person- years)	Incidence rate (/1,000 person-years)	Unadjusted		Model 1		Model 2	
					HR (95% CI)	*P*	HR (95% CI)	*P*	HR (95% CI)	*P*
Healthy Control	10,955	77	64,358.35	1.20	1 (Ref.)		1 (Ref.)		1 (Ref.)	
ESRD	10,955	180	53,507.48	3.36	2.83 (2.17–3.70)	< 0.0001	2.95 (2.26–3.85)	< 0.0001	1.43 (0.97–2.12)	0.07
KTR	10,955	200	62,107.74	3.22	2.69 (2.07–3.50)	< 0.0001	2.72 (2.09–3.54)	< 0.0001	1.41 (0.96–2.07)	0.08
ESRD	10,955	180	53,507.48	3.36	1 (Ref.)		1 (Ref.)		1 (Reference)	
KTR	10,955	200	62,107.74	3.22	0.95 (0.78–1.17)	0.65	0.928 (0.76–1.14)	0.47	1.02 (0.83–1.26)	0.84

Model 1: Cox proportional hazard (PH) regression model adjusted for age and sex.

Model 2: Cox proportional hazard (PH) regression model adjusted for age, sex, diabetes mellitus, hypertension, dyslipidemia, income, and Charlson comorbidity index.

*POAG* primary open-angle glaucoma, *HR* hazard ratio, *CI* confidence interval, *ESRD* end stage renal disease, *KTR* kidney transplantation recipients.

### PACG incidence in KTRs and ESRD patients

**Figure 2:**
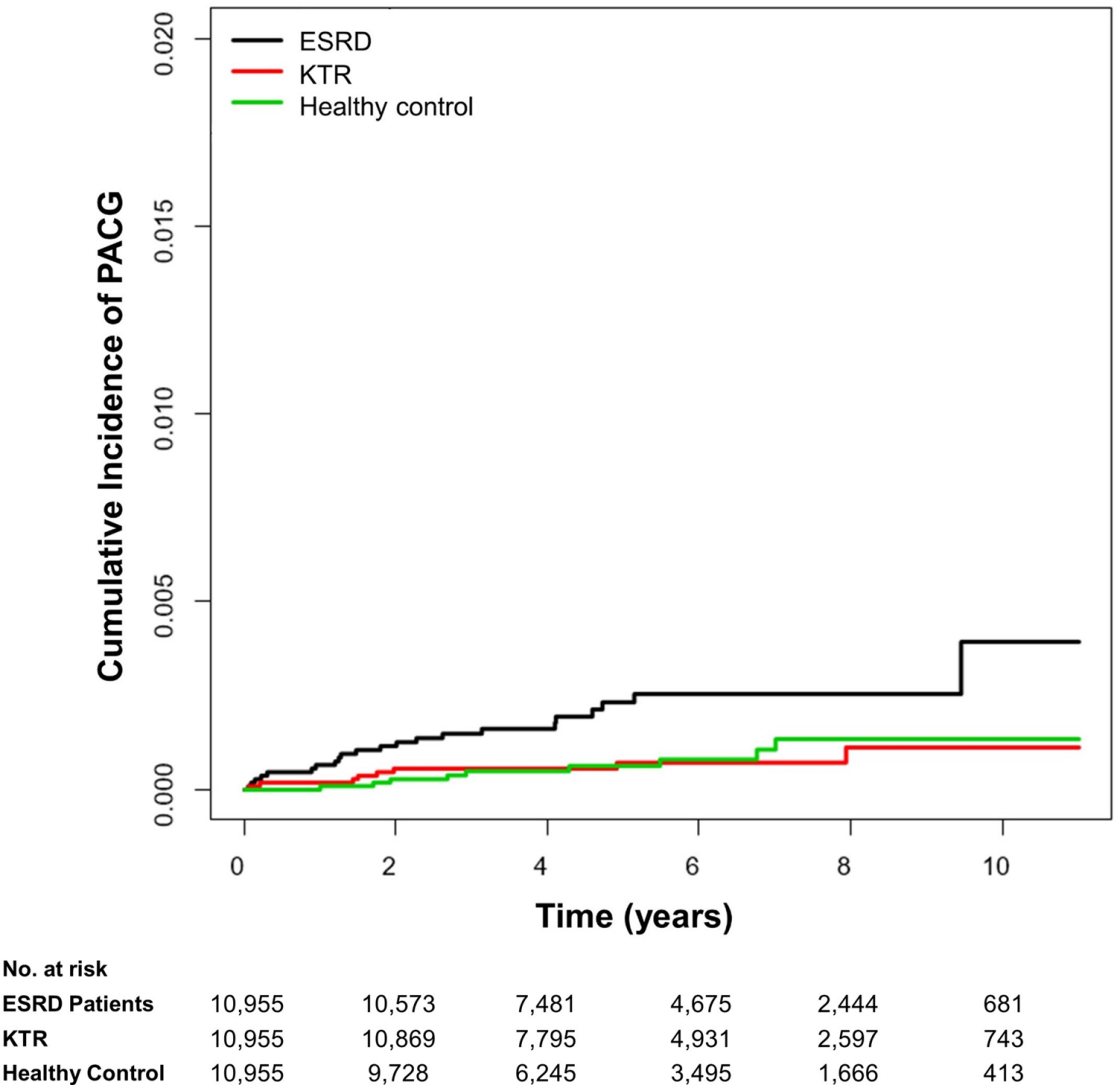
Cumulative Incidence of PACG in ESRD Patients, KTRs, and Healthy Controls. PACG incidence was significantly greater in ESRD patients (0.41/1,000 person-years) than in healthy controls (0.14/1,000 person-years, *P* = 0.008). However, there was no significant difference of PACG incidence rate between KTRs (0.13/1,000 person-years) and healthy controls (*P* = 0.86) when adjusted for age and sex (Model 1), or age, sex, DM, HTN, dyslipidemia, income, and CCI (Model 2). The PACG incidence rate was significantly lower in KTRs as compared to ESRD patients in both Model 1 (HR = 0.32, *P* = 0.006) and Model 2 (HR = 0.35, *P* = 0.02). *KTR* kidney transplant recipient, *KT* kidney transplant, *CKD* chronic kidney disease, *ESRD* end stage renal disease, *PACG* primary angle-closure glaucoma, *DM* diabetes mellitus, *HTN* hypertension, *CCI* Charlson comorbidity index, *HR* hazard ratio.

During the study period, a total of 39 (0.1%) patients were diagnosed with PACG. The incidence rate of PACG was significantly greater in ESRD patients (0.41/1,000 person-years) than in healthy controls (0.14/1,000 person-years, *P* = 0.008). However, there was no significant difference in PACG incidence rate between KTRs (0.13/1,000 person-years) and healthy controls (*P* = 0.86) (Fig. 2). This outcome remained unchanged even when the subjects were adjusted for age and sex (Model 1), or age, sex, DM, HTN, dyslipidemia, income, and CCI (Model 2) (Table 3). The PACG incidence rate was significantly lower in KTRs compared to ESRD patients in both Model 1 (HR = 0.32, *P* = 0.006) and Model 2 (HR = 0.35, *P* = 0.015). The numbers of subjects at risk of PACG among the study groups, stratified by time, are provided (Supplementary Table 3). The subgroup analysis according to the confounding variables including age, sex, DM, HTN, dyslipidemia, and duration of dialysis are provided in Supplementary Table 4.

**Table 3:** PACG incidence in ESRD patients, KTRs, and healthy controls.

Group	N	PCAG incidence	Duration (person- years)	Incidence rate (/1000 person-years)	Unadjusted		Model 1		Model 2	
					HR (95% CI)	*P*	HR (95% CI)	*P*	HR (95% CI)	*P*
Healthy Control	10,955	9	64,358.35	0.14	1 (Ref.)		1 (Ref.)		1 (Ref.)	
ESRD	10,955	22	53,507.48	0.41	2.84 (1.31–6.18)	0.008	3.00 (1.38–6.52)	0.006	3.50 (1.08–11.31)	0.036
KTR	10,955	8	62,107.74	0.13	0.92 (0.35–2.38)	0.86	0.93 (0.36–2.42)	0.89	1.20 (0.33–4.40)	0.78
ESRD	10,955	22	53,507.48	0.41	1 (Ref.)		1 (Ref.)		1 (Ref.)	
KTR	10,955	8	62,107.74	0.13	0.95 (0.78–1.17)	0.65	0.32 (0.14–0.72)	0.006	0.35 (0.15–0.82)	0.015

Model 1: Cox proportional hazard (PH) regression model adjusted for age and sex.

Model 2: Cox proportional hazard (PH) regression model adjusted for age, sex, diabetes mellitus, hypertension, dyslipidemia, income, and Charlson comorbidity index.

*PACG* primary angle-closure glaucoma, *HR* hazard ratio, *CI* confidence interval, *ESRD* end stage renal disease, *KTR* kidney transplantation recipients.

## Discussion

In this study, we investigated the nationwide population-based incidence of POAG and PACG in ESRD patients and KTRs and compared it with that of subjects with healthy kidney function. The present study results demonstrated that POAG risk was nominally increased in ESRD patients but did not reach statistical significance after controlling for multiple confounding factors. Although KT did not affect the risk of developing POAG, it significantly reduced the risk of developing PACG, which was significantly increased in ESRD patients as compared to healthy controls.

CKD and major eye diseases are assumed to share common risk factors, including age, smoking, DM, HTN, obesity, and dyslipidemia. Moreover, it is well known that the risk of vision threatening diseases such as age-related macular degeneration and diabetic retinopathy increases in CKD patients^16,17^. However, whether the risk of developing POAG in CKD patients increases remains controversial. A study of 3,280 Malaysian adults reported that IOP was higher in CKD patients than in those without CKD; however, the study reported no association between CKD and glaucoma^8^. Another study from the Korea National Health and Nutrition Examination Survey (2010–2011) demonstrated no association of CKD with POAG, but low estimated glomerular filtration rate (< 45 ml/min/1.73 m^2^) was significantly associated with POAG^7^. A recent US National Health and Nutrition Examination Survey (2005–2008) study also revealed no association of POAG with CKD^9^. A study from the Asian Eye Epidemiology Consortium, which included 28,925 participants, concluded that the association between CKD and POAG may only be present specifically in East Asians (Korean and Chinese), but not in the overall Asian population^18^.

Our data revealed that POAG risk was not significantly high even in ESRD patients with deteriorated renal function when adjusted for multiple confounding factors. The present finding may imply that the nominally increased prevalence of POAG in ESRD patients may be attributed to the several shared confounding risk factors between ESRD and POAG, including HTN, DM, dyslipidemia, income, and comorbid systemic diseases. Furthermore, KT did not alter POAG risk in KTRs as compared to that in ESRD patients. Approximately 30% of KTRs are known to experience acute kidney transplant rejection^19,20^, and the mainstay of treatment for graft rejection is high-dose steroid pulse therapy^21^. In addition, almost all KTRs in Korea are prescribed low dose corticosteroids as a maintenance therapy^21,22^. Given the common use of steroids in KTRs, the POAG risk may not be reduced in this population despite improved renal function and prevention of possible ophthalmic complications induced by dialysis, such as BP and IOP fluctuation, and subsequent decrease in ocular perfusion pressure^11^.

The reported effect of hemodialysis on IOP in the existing literature is inconsistent: the IOP can rise, decrease, or may not change^23^. Hemodialysis can cause complex hemodynamic changes in the ocular structure. During hemodialysis, the rapid decrease in plasma osmolality and relative increase in intracellular urea concentration results in a gradient between plasma and ocular compartments^24^. This induces a shift of extracellular fluid from the blood to the anterior chamber, which eventually increases IOP. In contrast, fluid removal during ultrafiltration, without concomitant albumin removal, increases colloid osmotic pressure^25^. This causes an influx of fluid from the aqueous humor to the plasma, thereby reducing IOP. The dynamic effect of hemodialysis on IOP may be dependent on hemodialysis techniques as well as the subjects’ ethnicity or aqueous outflow facility^11,26,27^.

The present results revealed that PACG risk was significantly greater in ESRD patients. Hemodialysis can alter the angle structure or anterior chamber depth (ACD). Rever et al.^27^ reported significant decrease of ACD during acetate hemodialysis, but not with bicarbonate hemodialysis. Another study that used ultrasonic biometry or anterior segment-optical coherence tomography also reported a significant decrease in ACD^12,14^. Previous findings that report an excessive rise in IOP during or after hemodialysis in eyes with narrow angle support the notion of elevated risk of PACG in ESRD patients^28–30^. Following this idea, our data further showed that KT significantly reduces PACG risk. Periodic avoidance of hemodialysis in KTRs might have contributed to the lower risk of PACG relative to that observed in healthy controls. However, as the present study is limited by its non-randomized and retrospective design, a further prospective longitudinal study is warranted to reach a confirmative conclusion.

The present study has the following limitations. First, the diagnosis of POAG and PACG relied on ICD-10 codes, and glaucoma-related ophthalmic parameters such as IOP or visual field parameters or glaucoma-related medication or procedures were not obtained. It may be argued that the ICD-10 code-dependent diagnosis may lead to inaccurate diagnosis of POAG and PACG. However, the overall incidence of POAG and PACG in this study was quite similar to that of previous epidemiological studies^31–33^. This may alleviate concerns about code-based diagnosis and study enrollment. Second, a history of cataract surgery or vitrectomy, which can alter the risk of PACG, was not considered in the evaluation of PACG risk. As lateral information is absent in the code-dependent setting, it was prohibitively impossible to match the laterality of PACG diagnosis and history of cataract surgery or vitrectomy. Third, the diagnosis of CKD was determined based on ICD-10 codes, and the eGFR levels were not obtained in this study population. Therefore, we could not further investigate the risk of glaucoma according to the eGFR levels. Lastly, the present study included a large number of populations matched for various confounding factors including age and sex. However, one disadvantage of this matching is that the study population may be restricted to a particular number of individuals matched on specific variables. Further analyses, such as the use of polynomial scores, can improve data interpretation.

In conclusion, the present nationwide population-based cohort study showed that there was no significant association of POAG incidence risk in ESRD patients and KTRs after controlling for multiple confounding factors. However, the PACG risk was significantly increased in ESRD patients. Interestingly, KT reduced the risk of PACG in ESRD patients to a level similar to that in healthy controls. Thus, it would be prudent to monitor the onset of PACG in high-risk ESRD patients. Furthermore, as ESRD and POAG share common risk factors, it is important to monitor ESRD patients for the development of POAG.

## Methods

This study was undertaken as a part of RESTORE (REnal tranSplanT and OculaR disEases study) project—a retrospective, longitudinal cohort study based on the National Health Insurance Service (NHIS) database. The primary objective of the RESTORE project was to investigate the effect of KT on the national population-based incidence and prognosis of various eye diseases, including age-related macular degeneration, retinal vein occlusion, and glaucoma.

The present study was approved by the Institutional Review Board (IRB) of Seoul National University Hospital (SNUH, IRB No.: E-1906–046-1038). Access to the NHIS database was approved by ministry of Health and Social Affairs of Korea government. Requirement for informed consent was waived because of the retrospective study design and absence of any additional medical intervention on the study participants. The study was conducted as per tenets of the latest version of the Declaration of Helsinki.

### Data source

Republic of Korea has a compulsory single-payer health insurance system, which is managed by the NHIS. All healthcare providers are required to submit medical claims with information, including demographics, diagnostic codes based on the International Classification of Diseases (ICD)‐10 codes^34^, procedure codes, prescription records, and healthcare facilities, to NHIS for review and reimbursement. Therefore, the NHIS claims system acts as a centralized database structure and provides a nationwide, population-based data source.

In this study, the subjects were recruited from the NHIS database from 2007 to 2015 (*n* = 47,516,098) and evaluated for clinical information such as demographics, insured medical services, and disease diagnosis in subjects. The ICD-10 codes were used to define the comorbidities and causes of kidney disease. During the study period, KT was newly performed in 13,179 recipients who were identified by the ICD-10 codes R3280 (KT) or V005 (KT related treatment, V code for Korean rare incurable diseases). In Korea, these patients routinely take postoperative immunosuppressants and corticosteroids throughout their life as a maintenance therapy to prevent graft rejection^21,22^. ESRD patients were filtered from the dataset based on CKD diagnosis (N18-19) and history of dialysis for more than 3 months (Z49, Z99.2, and O7011-7020 [hemodialysis] or O7071-7075, and V003 [peritoneal dialysis]). Patients with ESRD were 1:1 matched with KTRs for age, sex, duration of renal replacement therapy, and history of underlying HTN and DM. Healthy controls without any history of CKD were also 1:1 matched with KTRs for age, sex, and year of inclusion.

The medication history of subjects obtained from the NHIS database and ICD-10 codes were used to identify the comorbidities. Patients with ICD-10 codes of HTN (I10-I15, I159, I151, and I1528) or those with a history of antihypertensive medication use for more than two times within a year before KT were considered to have HTN. Patients with ICD-10 codes of DM (E109, E119, E139, E149, E101, E111, E131, E141, E105, E115, E135, and E145) or those with history of taking oral hypoglycemic agents or insulin were considered to have DM. Dyslipidemia was defined by the ICD-10 code of dyslipidemia (E78) or medication history of taking lipid lowering agents.

The diagnosis of POAG or PACG was confirmed when a diagnosis of ICD-10 codes, H40.1 or H40.2, respectively, was made at least three times within one year. H40.1 included unspecified open-angle glaucoma (H4010), POAG (H4011), low-tension glaucoma (H4012), pigmentary glaucoma (H4013), capsular glaucoma with pseudo exfoliation of lens (H4014), and residual stages of open-angle glaucoma (H4015). H40.2 included unspecified primary angle-closure glaucoma (H4020), acute angle-closure glaucoma (H4021), chronic angle-closure glaucoma (H4022), intermittent angle-closure glaucoma (H4023), and residual stage of angle-closure glaucoma (H4024).

The present study excluded subjects based on the following criteria: (1) KT recipients (KTRs) who were not matched to ESRD patients (*n* = 825), (2) subjects with a history of glaucoma before enrollment (*n* = 1,018), (3) subjects with a history of multiple organ transplantations (*n* = 196), or (4) subjects younger than 19 years (*n* = 185). Finally, an equal number of KTRs, ESRD patients, and healthy controls (total, 32,865 subjects) were enrolled in the present study (Supplementary Fig. 1).

### Data collection

Demographic data, including age, sex, income level, dialysis modality, duration of dialysis, date of KT, medications used for induction therapy, history of desensitization, and Charlson comorbidity index (CCI), were obtained from the NHIS database. CCI is a useful indicator for assessing the severity of a patient's comorbidity. The CCI was modified to four categories (0, 1–2, 3–4, and ≥ 5) to adjust subjects’ current status: 1-year mortality rate of 12% for "0"; 26% for "1–2"; 52% for "3–4"; 85% for " ≥ 5."^35^ During the study period, information of immunosuppressant use, such as tacrolimus, cyclosporine, and corticosteroids, were collected and analyzed.

### Statistical analysis

All statistical analyses were performed using the SAS 9.4 program (SAS Institute, Cary, NC, USA). The data of baseline characteristics are provided as mean ± standard deviation, median with interquartile range, numbers, or as numbers with percentage (%). Chi-square test was applied for categorical variables, and analysis of variance for continuous variables. The Kruskal–Wallis test was used to analyze the continuous variables failed to show normality. The primary outcome of the present study was the incidence of POAG and PACG among the study population. Subjects were followed until the diagnosis of glaucoma (POAG or PACG), or on December 31, 2017. The incidence of glaucoma was presented as events per 1,000 person-years. A Cox proportional hazard regression model was used to calculate the hazard ratios (HR) for glaucoma incidence, first with a univariate analysis, and then with two multivariable analysis models (Model 1: adjusted model for age and sex; Model 2: adjusted for age, sex, DM, HTN, dyslipidemia, income, and CCI). Kaplan–Meier curves were plotted and compared using the log-rank test. Statistical significance was determined at the level of *P* < 0.05.

### Ethics approval

The present study was approved by the Institutional Review Board (IRB) of Seoul National University Hospital (SNUH, IRB No.: E-1906-046-1038). Requirement for informed consent was waived because of the retrospective study design and absence of any additional medical intervention on the study participants. The study was conducted as per tenets of the latest version of the Declaration of Helsinki.

## Supplementary Information

Supplementary Information

## Data Availability

The datasets generated and/or analyzed in the current study are available from the corresponding author on reasonable request.

## References

[1] Quigley HA, Broman AT (2006). The number of people with glaucoma worldwide in 2010 and 2020. Br. J. Ophthalmol..

[2] Tham YC (2014). Global prevalence of glaucoma and projections of glaucoma burden through 2040: a systematic review and meta-analysis. Ophthalmology.

[3] Chan EW (2016). Glaucoma in Asia: regional prevalence variations and future projections. Br. J. Ophthalmol..

[4] Chen TK, Knicely DH, Grams ME (2019). Chronic kidney disease diagnosis and management: a review. JAMA.

[5] Wong CW, Wong TY, Cheng CY, Sabanayagam C (2014). Kidney and eye diseases: common risk factors, etiological mechanisms, and pathways. Kidney Int..

[6] Gao B (2011). Ocular fundus pathology and chronic kidney disease in a Chinese population. BMC Nephrol..

[7] Shim SH (2016). Association between renal function and open-angle glaucoma: the Korea National Health and Nutrition Examination Survey 2010–2011. Ophthalmology.

[8] Nongpiur ME (2010). Chronic kidney disease and intraocular pressure: the Singapore Malay Eye Study. Ophthalmology.

[9] Zhu Z (2020). Visual impairment and major eye diseases in chronic kidney disease: the National Health and Nutrition Examination Survey, 2005–2008. Am. J. Ophthalmol..

[10] Chapman JR (2011). The consequences of successful transplantation. The Lancet.

[11] Hu J (2013). Effect of hemodialysis on intraocular pressure and ocular perfusion pressure. JAMA Ophthalmol..

[12] Gracitelli CP (2013). Anterior chamber depth during hemodialysis. Clin. Ophthalmol..

[13] Chen H, Zhang X, Shen X (2018). Ocular changes during hemodialysis in patients with end-stage renal disease. BMC Ophthalmol..

[14] Shin YU (2019). Effect of hemodialysis on anterior chamber angle measured by anterior segment optical coherence tomography. J. Ophthalmol..

[15] Olawoye OO, Ogunleye T, Sarimiye TF, Bello TO (2018). Acute angle closure following hemodialysis in a 34-year-old Nigerian female. Niger J. Clin. Pract..

[16] Deva R (2011). Vision-threatening retinal abnormalities in chronic kidney disease stages 3 to 5. Clin. J. Am. Soc. Nephrol..

[17] Nusinovici S, Sabanayagam C, Teo BW, Tan GSW, Wong TY (2019). Vision impairment in CKD patients: epidemiology, mechanisms, differential diagnoses, and prevention. Am. J. Kidney Dis..

[18] Tham YC (2020). Is kidney function associated with primary open-angle glaucoma? Findings from the Asian Eye epidemiology consortium. Br. J. Ophthalmol..

[19] Go J (2019). A half-century 3000 cases of kidney transplant experiences in a single hospital: the Longest Registry in Korea. Transplant Proc..

[20] Koo EH (2015). The impact of early and late acute rejection on graft survival in renal transplantation. Kidney Res. Clin. Pract..

[21] Kasiske BL (2010). KDIGO clinical practice guideline for the care of kidney transplant recipients: a summary. Kidney Int..

[22] Park S (2020). Characteristics of kidney transplantation recipients over time in South Korea. Korean J. Intern. Med..

[23] Levy J, Tovbin D, Lifshitz T, Zlotnik M, Tessler Z (2005). Intraocular pressure during haemodialysis: a review. Eye (Lond).

[24] Sitprija V, Holmes JH, Ellis PP (1964). Changes in intraocular pressure during hemodialysis. Invest. Ophthalmol..

[25] Tokuyama T, Ikeda T, Sato K (1998). Effect of plasma colloid osmotic pressure on intraocular pressure during haemodialysis. Br. J. Ophthalmol..

[26] Burn RA (1973). Intraocular pressure during haemodialysis. Br. J. Ophthalmol..

[27] Rever B, Fox L, Christensen R, Bar-Khayim Y, Nissenson AR (1983). Adverse ocular effects of acetate hemodialysis. Am. J. Nephrol..

[28] Jaeger P, Morisod L, Wauters JP, Faggioni R (1980). Prevention of glaucoma during hemodialysis by mannitol and acetazolamide. N. Engl. J. Med..

[29] Cecchin E, De Marchi S, Tesio F (1986). Intraocular pressure changes during hemodialysis. Nephron.

[30] De Marchi S, Cecchin E, Tesio F (1989). Intraocular pressure changes during hemodialysis: prevention of excessive dialytic rise and development of severe metabolic acidosis following acetazolamide therapy. Ren. Fail..

[31] Pan CW (2017). Longitudinal cohort study on the incidence of primary open-angle glaucoma in Bai Chinese. Am. J. Ophthalmol..

[32] Kreft D, Doblhammer G, Guthoff RF, Frech S (2019). Prevalence, incidence, and risk factors of primary open-angle glaucoma - a cohort study based on longitudinal data from a German public health insurance. BMC Public Health.

[33] Vijaya L (2013). Six-year incidence of angle-closure disease in a South Indian population: the Chennai Eye Disease Incidence Study. Am. J. Ophthalmol..

[34] Quan H (2005). Coding algorithms for defining comorbidities in ICD-9-CM and ICD-10 administrative data. Med. Care.

[35] Charlson ME, Pompei P, Ales KL, MacKenzie CR (1987). A new method of classifying prognostic comorbidity in longitudinal studies: development and validation. J. Chronic. Dis..

