# Supplementary material for: Nationwide Glaucoma incidence in end stage renal disease patients and kidney transplant recipients
Source: Sci Rep. 2021 Apr 1;11:7418. doi: 10.1038/s41598-021-86846-3 (PMC8017003; doi:10.1038/s41598-021-86846-3)
Supplement: Supplementary file 1 — Supplementary Information [file 41598_2021_86846_MOESM1_ESM.pdf]

Supplementary Online Content

Nationwide Glaucoma Incidence in End Stage Renal Disease Patients and Kidney Transplant Recipients

Jong Joo Moon, MD, PhD[1*], Yong Woo Kim, MD, PhD[2*], Baek-Lok Oh, MD, MBA[2], Kyungdo Han, PhD[3], Dong Ki Kim, MD, PhD[1], Kwon Wook Joo, MD, PhD[1], Yon Su Kim, MD, PhD[1], Ki Ho Park, MD, PhD[2], Hajeong Lee, MD, PhD[1], Yong Chul Kim, MD, PhD[1], Jin Wook Jeoung, MD, PhD[2**]**

[*] These two authors contributed equally as first authors.
[**] These two authors contributed equally as corresponding authors.

[1]Division of Nephrology, Department of Internal Medicine, Seoul National University Hospital, Seoul, Korea
[2]Department of Ophthalmology, Seoul National University Hospital, Seoul, Korea
[3]Department of Medical Statistics, College of Medicine, Catholic University of Korea, Korea

Supplementary Figure 1. Flowchart Showing the Study Population

Supplementary Table 1. Number at Risk of POAG by Time in ESRD Patients, KTRs, and Healthy Controls.

Supplementary Table 2. Subgroup Analysis for Risk of POAG

Supplementary Table 3. Number at Risk of PACG by Time in ESRD Patients, KTRs, and Healthy Controls.

Supplementary Table 4. Subgroup Analysis for Risk of PACG

These supplementary materials have been provided by the authors to give readers additional information about their work.

Supplementary Figure 1. Flowchart Showing the Study Population

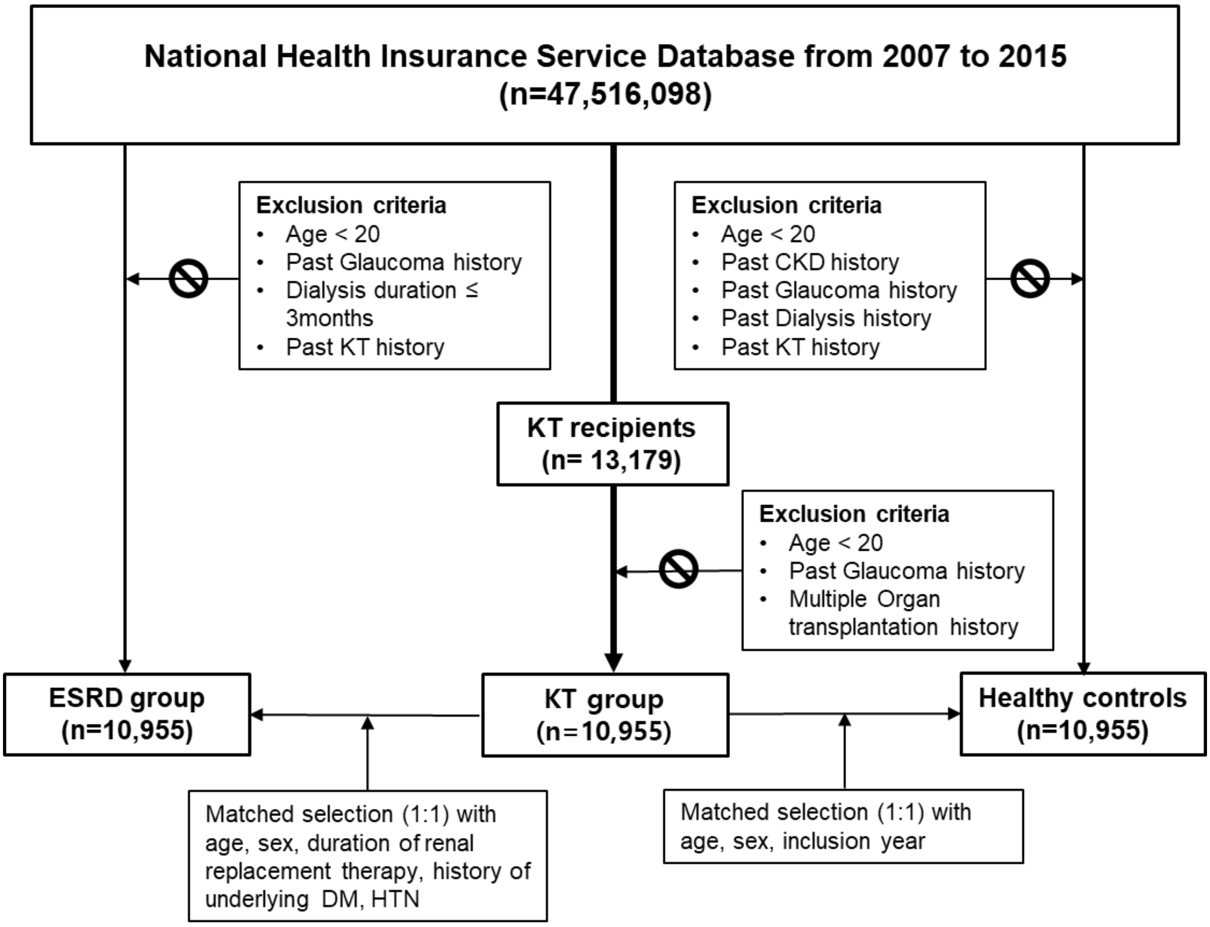

In this study, subjects were recruited from the NHIS database from the year 2007 to 2015 (n = 47,516,098). During the study period, 13,179 KTRs were identified by using the ICD-10 codes R3280 (KT) or V005 (KT related treatment, V code for Korean rare incurable diseases). ESRD patients were filtered with the diagnosis of CKD (N18-19), a more than 3-month history of dialysis (Z49, Z99.2, and O7011-7020 [hemodialysis] or O7071-7075, and V003 [peritoneal dialysis]). ESRD patients were matched with KTRs for age, sex, duration of renal replacement therapy, and history of underlying hypertension (HTN) and diabetes mellitus (DM). Healthy controls without any history of CKD were matched with KTRs for age, sex, and inclusion year. Subjects were excluded based on the following criteria: (1) KTRs who were not matched with ESRD patients (n = 825), (2) history of glaucoma before enrollment (n = 1,018), (3) history of multiple organ transplantations (n = 196), or (4)

subjects younger than 19 years (n = 185). Finally, an equal number of KTRs, ESRD patients, and healthy controls (total number of subjects, 32,865) were enrolled in the present study.

NHIS, National Health Insurance Service; KTR, kidney transplant recipient; KT, kidney transplant; CKD, chronic kidney disease; ESRD, end stage renal disease

Supplementary Table 1. Number at Risk of POAG by Time in ESRD Patients, KTRs, and Healthy Controls.

Group	Years											
	**0**	**1**	**2**	**3**	**4**	**5**	**6**	**7**	**8**	**9**	**10**	**11**
**ESRD**	10,955	10,691	10,573	9,008	7,481	6,079	4,675	3,412	2,444	1,523	681	11
**KTR**	10,955	10,908	10,869	9,308	7,795	6,365	4,931	3,602	2,597	1,641	743	11
**Healthy Control**	10,955	10,348	9,728	7,900	6,245	4,809	3,495	2,422	1,666	973	413	5

ESRD: End stage renal disease, KTR: Kidney transplantation recipients

Supplementary Table 2. Subgroup Analysis for Risk of POAG

	Group	*N*	Event	IR	HR (95% CI)	*P1*	*P2*	*P3*
**Age**						**0.007**		
**Age<40**	**ESRD**	**3092**	**46**	**2.62**	**3.51 (1.11–11.09)**		**0.008**	**0.021**
	**KTR**	**3092**	**26**	**1.34**	**1.84 (0.57–5.93)**			
	**Healthy**	**3092**	**7**	**0.35**	**1 (Ref.)**			
**40≤Age<65**	**ESRD**	**7580**	**129**	**3.68**	**1.17 (0.76–1.79)**		**0.008**	0.17
	**KTR**	**7580**	**165**	**3.98**	**1.33 (0.87–2.01)**			
	**Healthy**	**7580**	**68**	**1.57**	**1 (Ref.)**			
Age≥65	ESRD	283	5	5.61	2.77 (0.36–21.36)		0.53	0.70
	KTR	283	9	7.70	3.13 (0.44–22.45)			
	Healthy	283	2	1.54	1 (Ref.)			
Sex						0.45		
Male	ESRD	6484	114	3.69	1.43 (0.88–2.33)		0.26	0.59
	KTR	6484	136	3.75	1.49 (0.93–2.40)			
	Healthy	6484	54	1.44	1 (Ref.)			
Female	ESRD	4471	66	2.92	1.48 (0.77–2.86)		0.49	0.77
	KTR	4471	64	2.47	1.33 (0.69–2.56)			
	Healthy	4471	23	0.86	1 (Ref.)			
DM						0.21		
**No DM**	**ESRD**	**6539**	**78**	**2.27**	**2.00 (1.22–3.30)**		**0.012**	0.35
	**KTR**	**6539**	**90**	**2.38**	**2.11 (1.28–3.48)**			
	**Healthy**	**10263**	**62**	**1.02**	**1 (Ref.)**			
DM	ESRD	4416	102	5.34	0.80 (0.43–1.48)		0.68	0.80
	KTR	4416	110	4.52	0.77 (0.42–1.40)			
	Healthy	692	15	4.16	1 (Ref.)			
HTN						0.06		
**No HTN**	**ESRD**	**952**	**9**	**1.81**	**1.51 (0.61–3.71)**		**0.015**	**0.013**
	**KTR**	**952**	**21**	**3.87**	**3.05 (1.39–6.69)**			
	**Healthy**	**9159**	**54**	**0.99**	**1 (Ref.)**			
HTN	ESRD	10003	171	3.52	1.13 (0.70–1.83)		0.73	0.59
	KTR	10003	179	3.16	1.05 (0.65–1.83)			
	Healthy	1796	23	2.35	1 (Ref.)			
Dyslipidemia						0.17		
**No dyslipidemia**	**ESRD**	**6288**	**102**	**3.22**	**1.87 (1.13–3.08)**		**0.048**	0.99
	**KTR**	**4785**	**83**	**2.98**	**1.76 (1.05–2.93)**			
	**Healthy**	**9669**	**59**	**1.02**	**1 (Ref.)**			
Dyslipidemia	ESRD	4667	78	3.57	0.88 (0.48–1.62)		0.90	0.67
	KTR	6170	117	3.41	0.93 (0.52–1.66)			
	Healthy	1286	18	2.70	1 (Ref.)			
Dialysis duration						0.21		
< 5 years	ESRD	8496	148	3.55	1 (Ref.)		-	0.57
	KTR	8212	150	3.11	0.94 (0.74–1.18)			
	Healthy	-	-	-	-			
≥ 5 years	ESRD	2459	32	2.71	1 (Ref.)		-	
	KTR	2743	50	3.62	1.49 (0.94–2.35)			0.09
	Healthy	-	-	-	-			

P1, P-value for interaction with renal replacement therapy; P2, Comparison among the ESRD, KTR, and healthy groups; P3, Comparison between groups ESRD and KTR. POAG, primary open-angle glaucoma; IR, incidence rate; HR, hazard ratio; CI, confidence interval; ESRD, end-stage renal disease; KTR, kidney transplantation recipients; DM, diabetes mellitus; HTN, hypertension.

Supplementary Table 3. Number at Risk of PACG by Time in ESRD Patients, KTRs, and Healthy Controls.

Group	Years											
	**0**	**1**	**2**	**3**	**4**	**5**	**6**	**7**	**8**	**9**	**10**	**11**
**ESRD**	10,955	10,691	10,573	9,008	7,481	6,079	4,675	3,412	2,444	1,523	681	11
**KTR**	10,955	10,908	10,869	9,308	7,795	6,365	4,931	3,602	2,597	1,641	743	11
**Healthy Control**	10,955	10,348	9,728	7,900	6,245	4,809	3,495	2,422	1,666	973	413	5

ESRD: End stage renal disease, KT: Kidney transplantation

Supplementary Table 4. Subgroup Analysis for Risk of PACG

	Group	*N*	Event	IR	HR (95% CI)	*P1*	*P2*	*P3*
Age						0.86		
Age<40	ESRD	3092	2	0.11	3831694.9 (0–Inf)		>0.99	0.52
	KTR	3092	2	0.10	4116593.1 (0–Inf)			
	Healthy	3092	0	0	1 (Ref.)			
**40≤Age<65**	**ESRD**	**7580**	**20**	**0.57**	**3.54 (1.07–11.74)**		**0.008**	**0.009**
	**KTR**	**7580**	**6**	**0.14**	**0.99 (0.25–3.96)**			
	**Healthy**	**7580**	**9**	**0.21**	**1 (Ref.)**			
Age≥65	ESRD	283	0	0	-	-		-
	KTR	283	0	0	-			
	Healthy	283	0	0	-			
Sex						0.64		
**Male**	**ESRD**	**6484**	**11**	**0.36**	**5.69 (0.81–40.12)**		**0.043**	0.05
	**KTR**	**6484**	**3**	**0.08**	**1.46 (0.17–12.80)**			
	**Healthy**	**6484**	**3**	**0.08**	**1 (Ref.)**			
Female	ESRD	4471	11	0.49	2.80 (0.64–12.27)		0.18	0.12
	KTR	4471	5	0.19	1.21 (0.24–6.08)			
	Healthy	4471	6	0.22	1 (Ref.)			
DM						0.32		
**No DM**	**ESRD**	**6539**	**13**	**0.38**	**9.66 (2.33–40.06)**		**0.004**	0.11
	**KTR**	**6539**	**5**	**0.13**	**3.90 (0.79–19.29)**			
	**Healthy**	**10263**	**7**	**0.12**	**1 (Ref.)**			
DM	ESRD	4416	9	0.47	0.41 (0.06–2.72)		0.09	0.15
	KTR	4416	3	0.12	0.13 (0.02–0.99)			
	Healthy	692	2	0.55	1 (Ref.)			
**HTN**						0.13		
**No HTN**	**ESRD**	**952**	**3**	**0.60**	**34.64 (3.87–309.82)**		**0.005**	0.87
	**KTR**	**952**	**2**	**0.37**	**29.46 (2.51–345.32)**			
	**Healthy**	**9159**	**5**	**0.09**	**1 (Ref.)**			
**HTN**	**ESRD**	**10003**	**19**	**0.39**	**1.51 (0.42–5.40)**		**0.032**	**0.011**
	**KTR**	**10003**	**6**	**0.11**	**0.43 (0.10–1.80)**			
	**Healthy**	**1796**	**4**	**0.41**	**1 (Ref.)**			
Dyslipidemia						>0.99		
No dyslipidemia	ESRD	6288	12	0.38	3.85 (0.93–15.97)		0.05	0.14
	KTR	4785	3	0.11	1.15 (0.20–6.54)			
	Healthy	9669	9	0.16	1 (Ref.)			
Dyslipidemia	ESRD	4667	10	0.46	2631501.0 (0–Inf)		0.28	0.10
	KTR	6170	5	0.15	1078013.5 (0–Inf)			
	Healthy	1286	0	0	1 (Ref.)			
Dialysis duration						0.93		
**< 5 years**	**ESRD**	**8496**	**16**	**0.38**	**1 (Ref.)**		**-**	**0.040**
	**KTR**	**8212**	**6**	**0.12**	**0.37 (0.14–0.96)**			
	**Healthy**	**-**	**-**	**-**	**-**			
≥ 5 years	ESRD	2459	6	0.51	1 (Ref.)		-	0.30
	KTR	2743	2	0.14	0.41 (0.08–2.16)			
	Healthy	-	-	-	-			

P1, P-value for interaction with renal replacement therapy; P2, Comparison among the ESRD, KTR, and healthy groups; P3, Comparison between groups ESRD and KTR. POAG, primary open-angle glaucoma; IR, incidence rate; HR, hazard ratio; CI, confidence interval; ESRD, end-stage renal disease; KTR, kidney transplantation recipients; DM, diabetes mellitus; HTN, hypertension.
